# Pediatric posterior trans-olecranon fracture-dislocation of the elbow: a case report and review of literature

**DOI:** 10.3389/fsurg.2025.1487129

**Published:** 2025-08-05

**Authors:** Ren Xiang, Tang Qingsong, Zhao Kang, Hu Jie

**Affiliations:** Department of Orthopedics, Chengdu Women’s and Children’s Central Hospital, School of Medicine, University of Electronic Science and Technology of China, Chengdu, Sichuan, China

**Keywords:** elbow, trans-olecranon fracture, posterior dislocation, pediatrics, trauma

## Abstract

**Objective:**

Dislocations of the elbow are not common in skeletally immature patients. Herein, we present a case report on a rare pediatric posterior trans-olecranon fracture dislocation of the elbow, a type of dislocation that has never been reported in children. We aim to discuss the injury mechanism and introduce our treatment approach for this specific condition.

**Patient:**

A ten-year-old girl presented with pain, swelling and deformity of the left elbow following a fall from a rocking chair.

**Diagnoses and interventions:**

The complete injury history and detailed physical examination were recorded. Radiographs revealed fractures affecting the coronoid process of the ulna, olecranon of the ulna, radial neck, along with dislocation of radiocapitellar joint, and posterior medial displacement of proximal radial-ulnar joint. Based on these findings, the diagnosis of posterior trans-olecranon fracture dislocation of the elbow was made. To restore stability of the elbow, open reduction and internal fixation were performed. This involved securing the coronoid process of the ulna with the suture lasso technique and stabilizing the olecranon of the ulna with two 2.0 mm Kirschner wires inserted into the medullary cavity. The wires were subsequently removed at 2 months postoperatively.

**Outcomes:**

Three months after the initial surgery, which is one month after the removal of internal fixation, the affected elbow joint achieved a range of motion of 140° flexion and 0° extension, with no limitation in forearm rotation activities. The elbow joint was stable and painless during movement. At the one-year follow-up, no difference was observed in the function of the bilateral elbow joints, and imaging examinations showed normal anatomical relationships of the elbow joints.

**Conclusion:**

This specific type of injury, termed as posterior trans-olecranon fracture-dislocation of the elbow, is rare and has few reports in the pediatric population. We, hereby, report the case to emphasize the importance of promptly restoring the stability of the elbow and initiating early actively range-of-motion exercises to ensure a favorable outcome.

## Introduction

Dislocations of the elbow are not common in skeletally immature patients, accounting for only about 3%–6% of all elbow injuries in children (1). Based on various disruptions of the elbow joint structure and directions of dislocation, elbow joint dislocations can be categorized into distinct types, such as posterior dislocation, anterior dislocation, medial or lateral dislocation, divergent dislocation, and complex dislocation (2). Herein, we present a case report on a rare pediatric posterior trans-olecranon fracture dislocation of the elbow, a type of dislocation that has never been reported in children. We aim to discuss the injury mechanism and introduce our treatment approach for this specific condition.

## Case description

**Figure 1:**
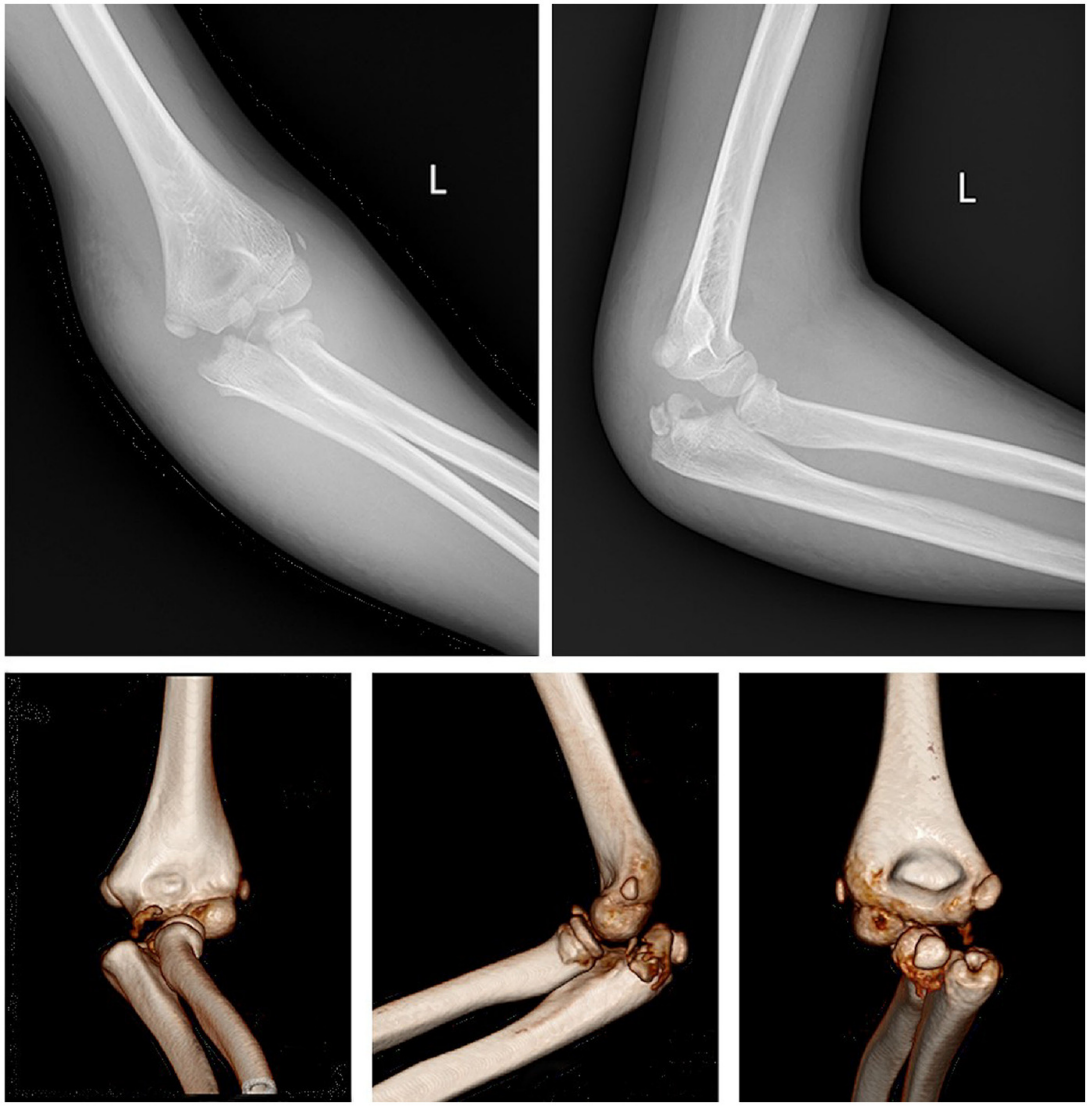
X-rays and CT scans showed fractures in the coronoid process, olecranon of the ulna, and the radial neck, along with dislocation of radiocapitellar joint and posterior medial displacement of proximal radial-ulnar joint.

**Figure 2:**
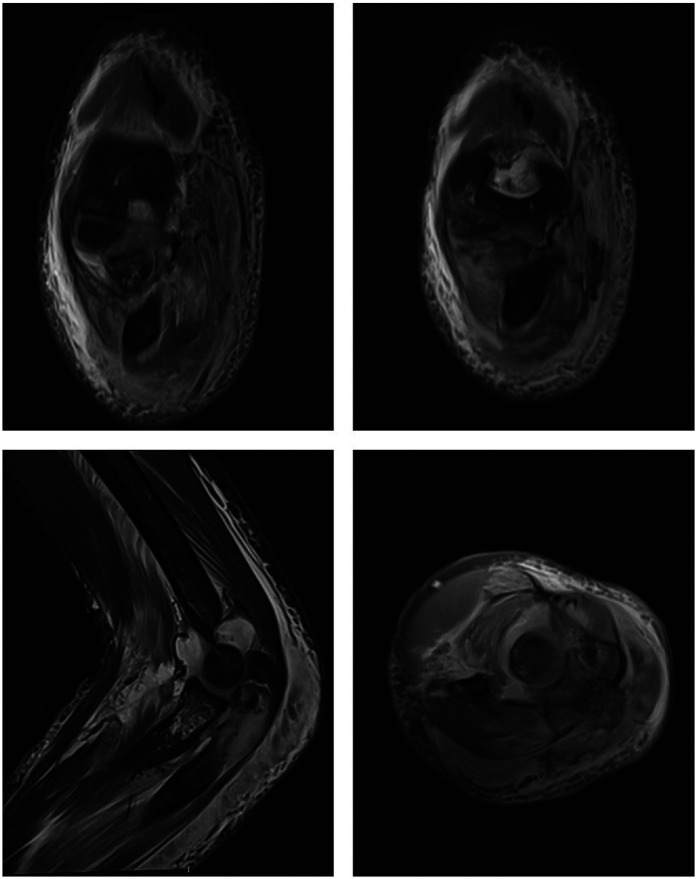
MRI showed tears in the attachment of the brachialis muscle, anterior joint capsule, and anterior periosteum of the proximal ulna, while the ligaments and soft tissues attached to the medial and lateral epicondyles of the humerus remained intact.

This study received ethical approval from the Ethics Committee of the Chengdu Women's and Children's Central Hospital (No.2023-102). Signed informed consent was obtained from the patient's guardians in accordance with the Declaration of Helsinki. A ten-year-old girl presented with pain, swelling and deformity of the left elbow following a fall from a rocking chair. The exact position of the injured limb at the time of the accident could not be accurately recalled by the patient. Clinical examination revealed significant restrictions in the flexion and extension movements of the left elbow joint, as well as limited rotation of the forearm. The skin of the left upper limb is intact without any ecchymosis. There was circular tenderness around the left elbow joint, and a bone rubbing sensation could be felt. The left shoulder joint, wrist joint, and fingers exhibited normal range of motion and function. The skin sensation in the injured limb showed no abnormalities. x-rays and CT scans revealed a complex pattern of injury, including fractures of the coronoid process and olecranon of the ulna, as well as the radial neck, accompanied by dislocation of radiocapitellar joint and posterior medial displacement of proximal radial-ulnar joint as a unit (Figure 1). MRI revealed tears in the attachment of the brachialis muscle, anterior joint capsule, and anterior periosteum of the proximal ulna, while the ligaments and soft tissues attached to the medial and lateral epicondyles of the humerus remained intact (Figure 2). Based on these findings, posterior transolecranon fracture-dislocation of the elbow was diagnosed.

**Figure 3:**
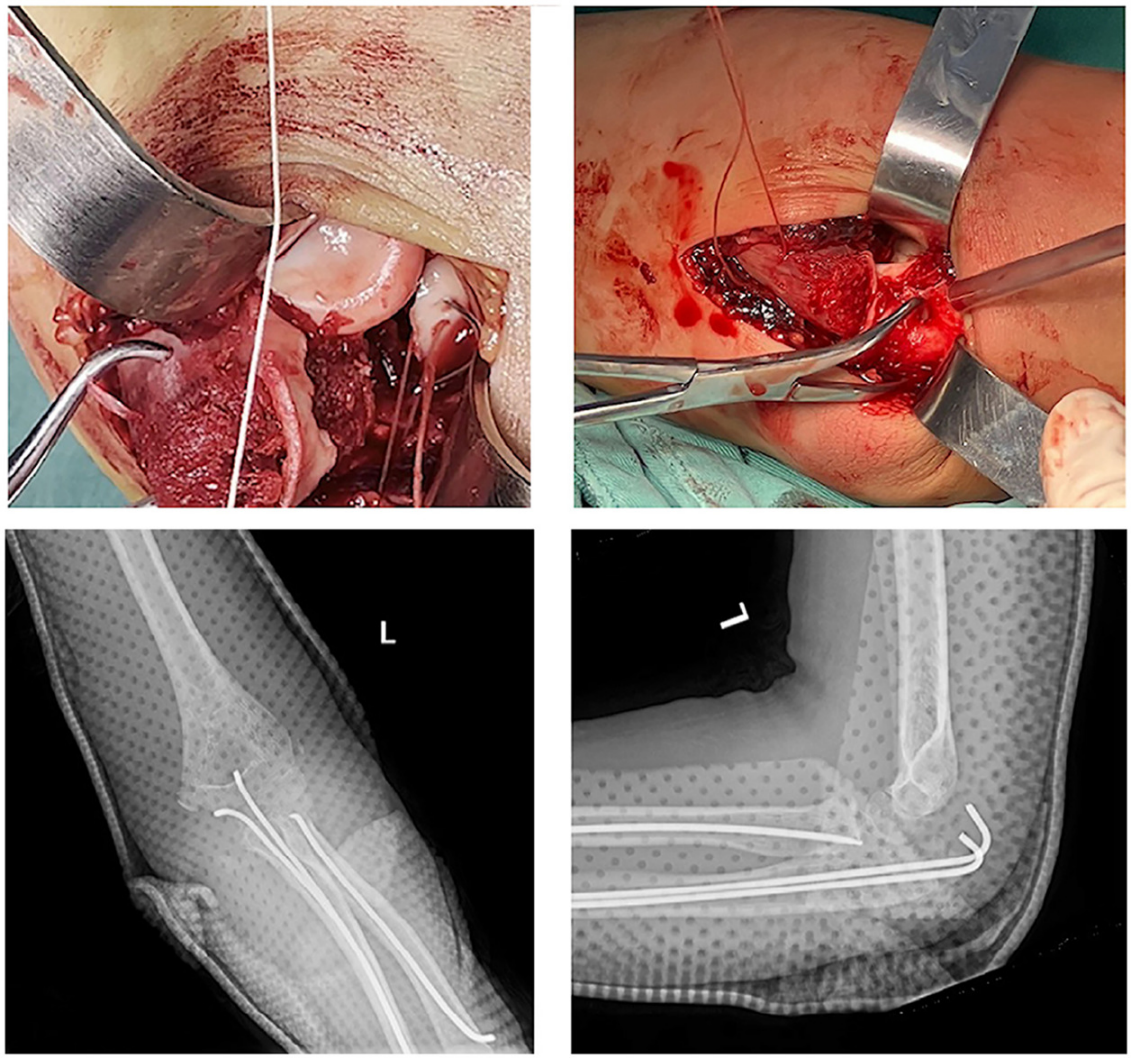
The coronoid process of the ulna fixed using the suture lasso technique and postoperative x-rays of the elbow joint.

Closed reduction was attempted, but it was unsuccessful. Therefore, open reduction and internal fixation was then performed under general anesthesia. The patient was positioned in the supine position with the arm abducted on the operating table. A pneumatic tourniquet was applied. After sterilization and draping, an incision was made posterior to the elbow, extending from the lateral epicondyle of the humerus to the proximal ridge of the ulna, utilizing the Boyd approach to expose the fractures. After clearing the surrounding hematoma, the proximal olecranon fragment was identified and turned over to expose the coronoid process and radius head. The olecranon metaphysis of the ulna suffered a transverse fracture, with the coronoid process fractured at its base, resulting in extensive damage to the articular surface of the olecranon and exposure of the subchondral bone. The coronoid process of the ulna was fixed using the suture lasso technique (3), with a 0-gauge absorbable suture (ETHICON VCPB1946H) passed through the coronoid fragment and then down through the medial and lateral bone tunnels in the coronoid fracture base, exiting directly posterior through the ulna (Figure 3). Subsequently, the olecranon fracture was reduced and stabilized with two 2.0mm Kirschner wires inserted intramedullary. With the elbow flexed to 90 degrees and the congruence of the humeroradial joint confirmed, the suture loops securing the coronoid fragment were tensioned and tied off. Finally, the radial neck fracture was reduced and fixed using a retrograde elastic nail technique (Figure 3). Following the fixation of the fractures, concentric reduction of the elbow was confirmed with an image intensifier and no instability was detected throughout the full range of motion. No ligament repair or reconstruction was deemed necessary.

**Figure 4:**
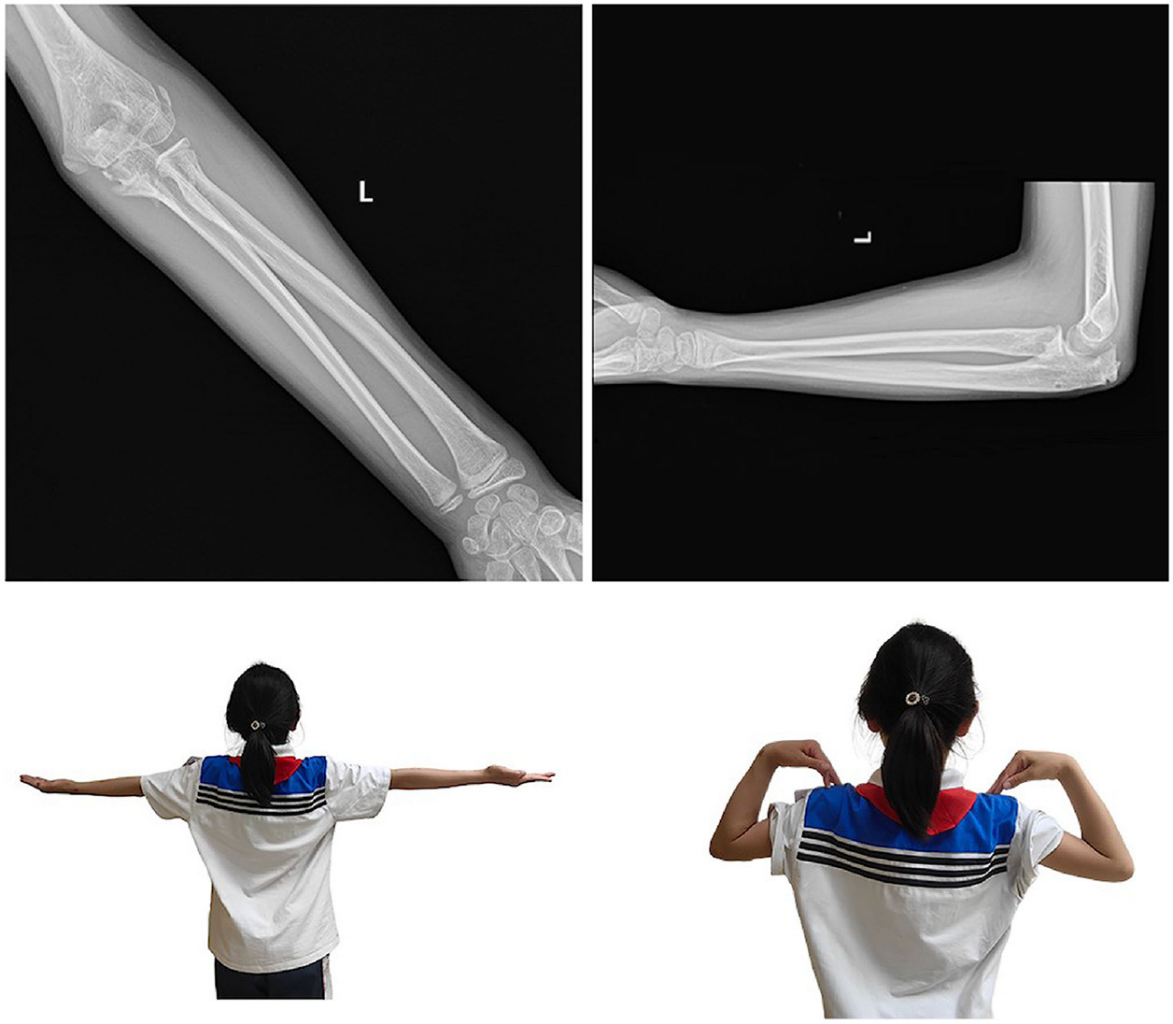
Postoperative x-rays at the one-year follow-up showed normal anatomical relationships of the elbow joints, and no functional difference was observed between the bilateral elbow joints.

Postoperatively, the injured arm was immobilized in a flexed elbow position at 90° with the forearm in neutral rotation for two weeks using a posterior splint. Following this, active flexion and extension exercises of the elbow were initiated under the protection of a hinged elbow brace, with a gradual increase of 20° per week for four weeks. No specific rehabilitation plan was implemented thereafter. The wires and elastic nails were removed two months postoperatively. In summary, the patient underwent surgery on the second day after the injury and was followed up at 2 weeks, 6 weeks, 2 months, 3 months, 6 months, and 1 year postoperatively, according to the scheduled timeline. Three months after the initial surgery, which is one month after the removal of internal fixation, the affected elbow joint achieved a range of motion of 140° flexion and 0° extension, with no limitation in forearm rotation activities. The elbow joint was stable and painless during movement. At the one-year follow-up, no difference was observed in the function of the bilateral elbow joints, and imaging examinations showed normal anatomical relationships of the elbow joints (Figure 4).

## Literature review

### Materials and methods

Relevant literature published up to May 2024 was retrieved from PubMed, Web of Science, and Embase databases. The search was conducted using keywords such as “elbow,” “elbow joint,” “elbow injuries,” “joint dislocation,” “olecranon fracture,” and “children/pediatric.” Only articles written in English were included in the retrieval. Additionally, we screened the references of each study to ensure that the majority of relevant studies were captured.

## Results

A total of three articles were retrieved, including one clinical study (4) and two reviews (5, 6). Notably, there were no case reports specifically focusing on children with the same type of condition.

### Etiology and diagnosis

Fracture of the olecranon of the ulna accompanied by posterior dislocation of the elbow joint is a rare injury. The key distinction between this type of injury and posterior Monteggia fracture lies in whether the anatomical relationship between the proximal radius and ulna is disrupted. When the olecranon fractures and the proximal radius and ulna move backward as a unit, it is diagnosed as posterior dislocation of the elbow joint through the olecranon (4).

The injury mainly affects the olecranon and coronoid process of the ulna, and the radial head fracture and dislocation. There is no consensus on whether this type of injury is always accompanied by lateral ligament complex injury.

Tao et al. (4) hypothesized that the injury mechanism of this type of fracture and dislocation is that the forearm is rotated posterolaterally relative to the humerus when an outstretched palm hit the ground. This high-energy impact initially causes an olecranon fracture, followed by a backward movement of the forearm. As a result, the distal humerus collides with the coronoid process, shearing off the radial head and ultimately leading to fractures.

### Treatment

Posterior dislocation of the elbow joint through an olecranon fracture is a severe injury. The primary treatment goals are to restore anatomical alignment, to regain stability, and to facilitate early functional activities. Tao et al. have emphasized the crucial role of ligament repair in restoring elbow joint stability, once the bony structure has been adequately addressed (4).

## Discussion

Elbow dislocations are uncommon in children, with the peak incidence typically occurring between the ages of 13 and 14, when the growth plates begin to fuse (5). Elbow joint fracture-dislocations represent a complex group of joint injuries that severely disrupt both the anatomic relationship and stability of the joint. Fractures associated with proximal radioulnar joint separation are categorized as Monteggia fractures or Monteggia-like injuries, which have received extensive attention and documentation in medical literature (6). Other frequent types of elbow joint dislocation often involve fractures of the medial and lateral condyles of the humerus. Trans-olecranon fracture-dislocations in children are exceedingly rare, with all reported cases involving anterior dislocation (7, 8). Notably, to date, there have been no reported instances of posterior trans-olecranon dislocations. In this report, we present a case of a posterior trans-olecranon fracture dislocation of the elbow in a ten-year-old girl who sustained an injury to her left elbow after falling from a rocking chair.

The injury mechanisms of elbow fracture-dislocations are intricate. Analyzing elbow injury structures through imaging aids in elucidating the injury mechanism and developing treatment plans based on the principle of reverse injury repair (9). In this case, the imaging revealed a transverse fracture at the metaphysis of the olecranon, along with fractures of the coronoid process and the radial neck. There were disruptures of the origin of the brachialis muscle, the anterior periosteum, and the anterior joint capsule of the elbow joint on the anterior proximal ulna. Additionally, there was posterior-medial displacement of the proximal radial-ulnar joint as a unit. These findings indicate that the posterior aspect of the metaphysis of the olecranon sustained considerable tension forces when the elbow joint was in a flexed position, leading to a transverse fracture at its midportion. Subsequently, the distal fragment displaced posteriorly, causing a collision between the coronoid process and the trochlea. This collision resulted in a fracture and tearing of the anterior soft tissue structures of the elbow. Simultaneously, a valgus deformity of the forearm occurred, leading to a fracture of the radial neck (10). The final presentation was a posterior trans-olecranon fracture-dislocation. The injury mechanism in this case differs significantly from that of anterior trans-olecranon fracture-dislocation, which involves anterior tensile stress on the proximal end of the ulna (11). It is also distinct from the posterior-lateral rotational injury mechanism associated with adult elbow terrible triad syndrome (12), and the hyperextension-valgus/varus injury mechanism related to elbow dislocations with medial and lateral condylar fractures (1, 13). This represents a distinct type of injury.

The ultimate treatment goal of the elbow fracture-dislocation is to restore full elbow motion without recurrent instability (5). In this particular case, addressing severe elbow fractures accompanied by dislocations poses a significant challenge: how to restore structural stability of the elbow, achieve optimal joint function, and simultaneously avoid exacerbating joint trauma. Here, concentric reduction and stability of the elbow were achieved through fracture fixation, without the necessity for ligament repair or reconstruction. Both preoperative imaging and intraoperative findings confirmed that, in contrast to adult elbow fracture-dislocations, pediatric elbow fracture-dislocations are primarily characterized by bony structural damage. Due to the successful restoration of joint stability, the injured arm was safely initiated into a gradual, active range of motion exercises, protected by a hinge splint, two weeks post-surgery. At the one-year follow-up, the outcome was highly satisfactory with no complications observed. The range of motion, appearance, and functionality of the affected arm were indistinguishable from those of the healthy arm.

## Conclusion

Pediatric posterior transolecranon fracture-dislocation of the elbow is a rare and specific form of elbow injury. A potential injury mechanism is the exertion of posterior tension and valgus stress on the elbow, when it is in a flexed position. The crucial aspects of treatment involve promptly achieving a concentric reduction of the elbow joint, while simultaneously identifying and addressing all associated injuries. Furthermore, initiating early functional activities is imperative for achieving optimal recovery outcomes.

## Data Availability

The original contributions presented in the study are included in the article/Supplementary Material, further inquiries can be directed to the corresponding author.
